# Metabolism of tRNAs and rRNAs shape immunoactive signatures in chronic obstructive pulmonary disease and pulmonary infections

**DOI:** 10.1016/j.omtn.2024.102298

**Published:** 2024-08-29

**Authors:** Zhenyi Hong, Xavier Bofill-De Ros

**Affiliations:** 1 Department of Molecular Biology and Genetics, Aarhus University, Aarhus, Denmark

## Main text

**Figure 1:**
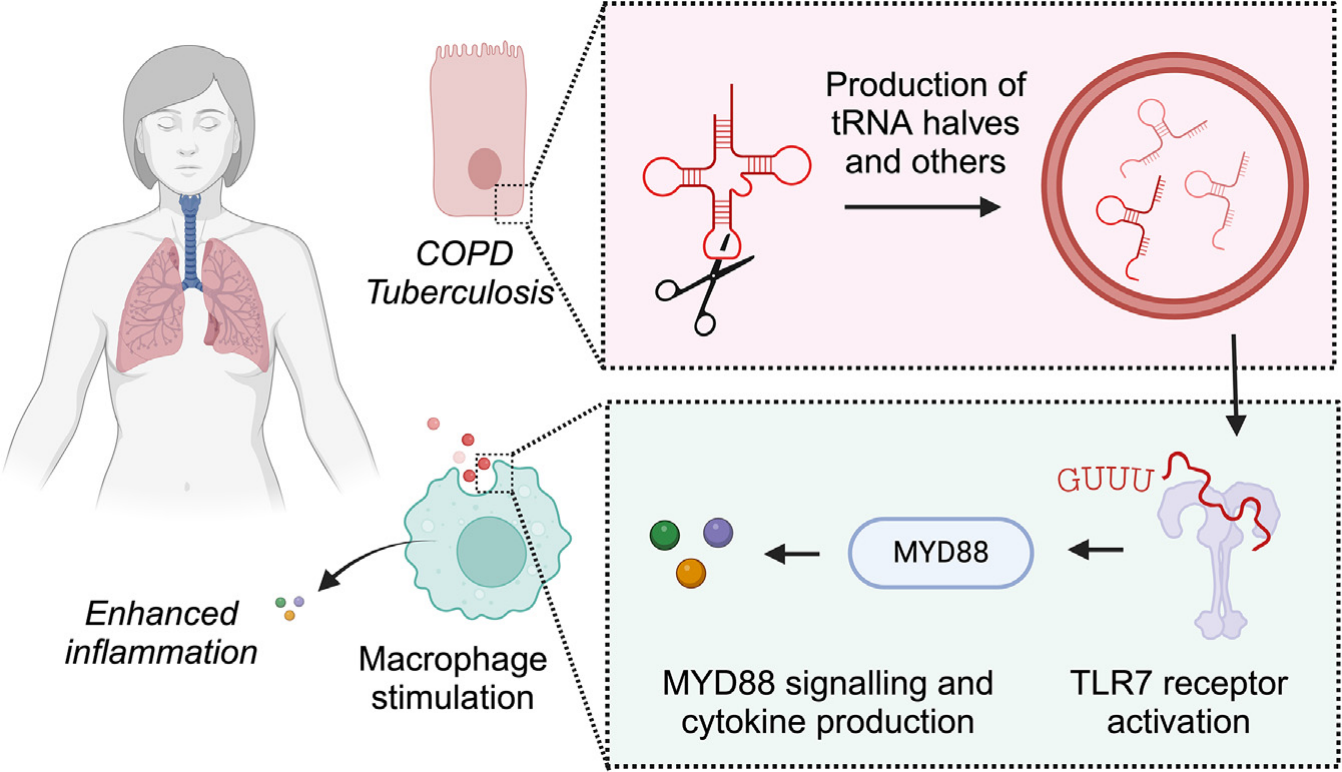
RNA fragments trigger immunoactive signatures in COPD and pulmonary infections via TLR7 The studies by Shigematsu et al. and Pawar et al. contribute to our understanding of how tRNA and rRNA fragments can contribute to prime the innate immune system. The authors show that 5′ tRNA^ValCAC^ and other small RNA fragments produced by cells in the context of COPD and *Mycobacterium tuberculosis* infection can be loaded to extracellular vesicles and stimulate the receiving macrophage by binding TLR7 in the endosomes. The illustration was created using BioRender.

In two recent studies from the Kirino lab, Shigematsu et al.^1^ and Pawar et al.^2^ show that certain fragments derived from the metabolism of tRNAs and rRNAs can act as potent endogenous activators of the innate immune system (Figure 1). This novel research ventures into the role of endogenous RNA fragments as modulators of innate immunity, providing insight into Toll-like receptor (TLR) 7 biology and potential therapeutic applications.

TLRs are key components of the innate immune system involved in the detection of pathogen-associated and damage-associated molecular patterns. This family of receptors can be found at the cell surface or in intracellular compartments such as the endosome. They are specialized in detecting ligands such as single-stranded RNAs (TLR7 and TLR8) or double-stranded RNAs (TLR3), which are usually associated with infection by pathogens but can also arise from endogenous cellular origins.^3^^,^^4^ Extracellular vesicles released from cells can be uptaken by macrophages and T and B cells and release their cargo into endosomes.^5^ Upon activation in the endosome, TLR7 promotes the transcription and induction of interferon and cytokines via the signaling cascade initiated by MyD88.

In these recent publications, the authors find that patients with chronic obstructive pulmonary disease (COPD) and pulmonary infections such as *Mycobacterium tuberculosis* present a distinct composition in tRNA and rRNA fragments when compared to healthy controls. To this end, the authors use a modified protocol for the preparation of next-generation sequencing libraries that consists of treatment with T4 polynucleotide kinase (PNK). This additional step, compared to traditional methods for cloning small RNAs such as microRNAs, allows the generation of 5′ phosphate and 3′ hydroxyl ends that are suitable for the adapter ligation and subsequent detection. Using this approach, the authors report that patients with COPD have an overexpression of 5′ tRNA^ValCAC^ and a reduction of 5′ tRNA^GlyGCC^. The same 5′ tRNA^ValCAC^ fragment was also found upregulated when human monocyte-derived macrophages were exposed to lipopolysaccharides and in the plasma of patients infected with *Mycobacterium tuberculosis*. This evidence indicates a role of 5′ tRNA^ValCAC^ as a maker of pulmonary inflammation. Similarly, the authors identified two ribosomal fragments derived from the 18S and 28S subunits differentially expressed in patients with COPD (18S-np22 and 28S-np4533, respectively).

Interestingly, functional studies of the role of fragment 5′ tRNA^ValCAC^ and rRNAs loaded into extracellular vesicles indicate their role as potent inducers of inflammatory cytokines such as tumor necrosis factor alpha, interleukin (IL)-1β, IL-12p40, and IL-6. The authors show that upon knockout of TLR7, the immunostimulatory activity of the identified tRNA and rRNA fragments is lost. In addition, this stimulation by tRNA and rRNA fragments provides derived macrophages with an increased capacity to eliminate bacteria in a cell-based assay.

In-depth molecular studies of 5′ tRNA^ValCAC^ and 5′ tRNA^HisGUG^, previously reported,^6^ indicate that the recognition of endogenous immunostimulant single-stranded RNAs by TLR7 is very specific. Through a series of mutagenesis studies, the authors identify that a terminal GUUU motif (or UUUG) is required for TLR7 activation. The authors note that even though both 5′ tRNA^ValCAC^ and 5′ tRNA^HisGUG^ retain a certain secondary structure, the identified GUUU motif is found in a terminal region on a single strand. This mechanism of recognition by TLR7 via the terminal GUUU motif appears to be conserved with other immunoregulatory RNAs such as miR-122-5p, miR-552-5p, or miR-548ah-5p. All of them when studied systematically presented similar 3-fold upregulation of inflammatory cytokines. Finally, the authors found that the GUUU motif can also activate TLR7-related cytokines when presented in the context of synthetic oligonucleotide sequences, thus indicating a robust recognition of such motifs. Lastly, the analysis of the effects of RNA modifications frequently found on tRNAs, such as pseudouridine and 2-methylguanosine, indicates that they can, to a moderate extent, affect the immunoregulatory role of 5′ tRNA^ValCAC^.

These two compelling studies from the Kirino lab also point to important questions for the field. It will be interesting to see how future studies address the role of the different enzymes involved in RNA metabolism in the formation of these immune regulators from endogenous RNA species, in particular whether angiogenin or other ribonucleases are responsible for the formation of tRNA halves upon COPD or other infectious or inflammatory processes. In this line, angiogenin was previously identified to generate stress-specific tRNA halves of specific tRNAs,^7^ including, among others, 5′ tRNA^ValCAC^. Other interesting aspects of the regulation of these immunoactive RNA species could be whether RNA ligases known to repair “nicked tRNAs” could play a role in regulating the production and release of these RNA fragments into biofluids.^8^^,^^9^

Overall, Shigematsu et al.^1^ and Pawar et al.^2^ show the role of specific tRNAs and rRNAs as biomarkers and immunoactive signatures. By leveraging novel library preparation methods, the authors exemplify the importance of incorporating cutting-edge methods for the detection of novel biomarkers in biologically relevant samples. The mechanistic work indicates that the activity of 5′ tRNA^ValCAC^ in macrophages has major effects by triggering the activity of TLR7. The in-depth mechanisms of biogenesis and export of these immunoactive products, as well as its effects in the context of living organisms, remain to be studied. Nevertheless, the authors presented a compelling case supporting the role of tRNA and rRNA fragments in the lungs of patients with COPD and bacterial infections.
